# Heterozygosity testing and multiplex DNA panel screening as a potential tool to monitor health and inbreeding in a small, closed dog population

**DOI:** 10.1186/s40575-018-0068-6

**Published:** 2018-12-28

**Authors:** S. F. A. Keijser, H. Fieten, M. Vos-Loohuis, C. J. Piek, H. Anderson, J. Donner, I. Scholten, M. Nielen, J. W. Hesselink, F. G. van Steenbeek

**Affiliations:** 1 Expertise Centre Genetics of Companion Animals, Department of Clinical Sciences of Companion Animals, Faculty of Veterinary Medicine, Utrecht University, Yalelaan 108, 3584 CM Utrecht, The Netherlands; 2 Department of Clinical Sciences of Companion Animals, Faculty of Veterinary Medicine, Utrecht University, Yalelaan 108, 3584 CM Utrecht, The Netherlands; 3 Genoscoper Laboratories Oy, P.O. Box 1040, 00251 Helsinki, Finland; 4 Dutch Shepherd Dog Club, Vijfsprongweg 126, 6741 JC Lunteren, The Netherlands; 5 Department of Farm Animal Health, Faculty of Veterinary Medicine, Utrecht University, Yalelaan 107, 3584 CM Utrecht, The Netherlands

**Keywords:** Canine health, Heterozygosity, Genetic disorder, Dog breeding, MyDogDNA™, Dutch shepherd dog, Von Willebrand’s disease

## Abstract

**Background:**

Selective breeding in populations with a limited effective population size may result in a loss of genetic diversity, which can cause an increased concentration of specific disease liability genes. The Dutch Shepherd Dog (DSD) in the Netherlands is an example of such a breed with a small effective population.

**Objective:**

To evaluate the measurement of genetic diversity and multiplex DNA panel screening for implementation in a breeding strategy for the Dutch Shepherd Dog (DSD) and to investigate the clinical relevance of potentially identified mutations in the multiplex DNA panel screening.

**Results:**

Genome-wide SNP testing showed genetic isolation and reduced genetic diversity within coat variety subgroups of the DSD. Panel screening identified a Von Willebrand’s Disease type I mutation. Although decreased Von Willebrand’s Factor proteins were significantly lower in DSDs carrying the VWD-I allele compared to the wildtype, clinical follow-up did not show a significant association between the clinical phenotype and VWD-I genotype.

**Conclusions:**

Genetic relationship measurement within a breed population may be a useful tool to enable breeding strategies to conserve genetic diversity. Results from a disease panel screening need to be evaluated for clinical relevance before breed selection restrictions can be considered.

**Electronic supplementary material:**

The online version of this article (10.1186/s40575-018-0068-6) contains supplementary material, which is available to authorized users.

## Plain english summary

Breed-related health issues in dogs are in part due to selective breeding. In effect, if only a small part of the population is allowed to breed, the individuals of that population become more and more alike, even at the level of genes. This is called limited genetic diversity, and it may increase the occurrence of genetic disorders. In contrast, populations that are not allowed to breed with each other become genetically different.

In this paper, we show the use of two genetic tools which support a sensible breeding strategy: 1) genetic relationships and diversity, and 2) screening test for genetic disorders. Both tools are provided by MyDogDNA™. As an example, these genetic tools were studied in the Dutch Shepherd Dog, a breed with three coat varieties (short, long, and wire haired). Historically, dogs with different coat varieties were not allowed to breed with each other.

The genetic relationship and diversity test showed that the three coat varieties of the Dutch Shepherd Dog were genetically isolated from each other, but within each group they were all much alike.

The screening test for genetic disorders in the Dutch Shepherd Dog showed the presence of the disease allele for Von Willebrand’s Disease type I, an inherited bleeding disorder. Further research showed that only a small percentage of the Dutch Shepherd Dogs were genetic carriers of this disease, and none showed evincive clinical signs.

In this study we found that genetic relationship results can show how far apart (sub) populations are, and what breeding strategies may be used to increase the diversity within subgroups. Furthermore, a screening test for genetic disorders can identify unexpected mutations that may cause clinical disease.

We conclude that a sensible breeding programme includes genetic tools, as well as individual and population-based clinical screening for disorders.

## Background

Dog breeds are known to be subject to human-induced limitations of the gene pool such as a popular sire effect and a breed barrier – a dog can only be registered as a certain breed if both parents are registered as such – resulting in reproductive isolation. Consequently, dogs from the same breed are genetically similar to each other [1], to such an extent that the breed can often be assessed by genotype alone, indicating genetic isolation between breeds [2]. Demographic models have shown that a small, effective population size and genetic bottlenecks may have a major effect on the spread of genome changes through a population, where deleterious mutations may result in genetic disorders in later generations [1, 3]. In small dog breed populations with a limited gene pool, such as the Dutch Shepherd Dog^1^ (DSD) population, an active approach to breeding healthy individuals is warranted to maintain genetic diversity for the future.

The DSD belongs to the shepherd dog type that originated in the Netherlands in the nineteenth century. It is grouped with e.g. the Saarloos wolfdog [4], and is a medium sized breed, measuring 55–62 cm high, and weighing between 23 and 28 kg. The DSD has an estimated population of approx. 2400 individuals in the Netherlands, with an assumed life expectancy of 11 years (estimated by the Dutch Shepherd Dog Club). The DSD population size is roughly thirty times lower than that of the Labrador retriever, which is the most popular breed in the Netherlands. The DSD has three coat varieties (short, long, and wire haired), which historically were not allowed to breed, although limited crossbreeding has been allowed since October 1st 2014 [5, 6]: the guidelines of the Fédération Cynologique International [7] still do not allow crossbreeding between long haired and wire haired varieties as coat issues such as felting would occur. Previous information on DSD health showed no indication of an increased predisposition to any genetic diseases [8–11]. However, the DSD population is thought to have limited genetic diversity, which harbours the risk of health issues related to inbreeding depression or increase of recessive disease in the future [12].

Genetic heterozygosity testing is currently routinely based on single nucleotide polymorphism (SNP) genotyping. SNP data can be used to test the genetic relationships of individuals and the genetic diversity of a population [13]. Heterozygosity is associated with an increase in e.g. cognition and memory [14, 15], thus shaping a population with the ability to respond to changing circumstances [12, 16]. A larger population size provides a greater predicted genetic diversity [17, 18].

Genome-wide SNP testing offers more accurate genetic diversity estimates than pedigree records or short-tandem repeat molecular markers [19] and the release of the canine genome sequence [20] facilitated an increase in research into genetic disorders [21, 22]. The development and availability of genomic tools has increased over the past two decades, allowing for more elaborate and precise testing in the future [23]. One of the possible tools is the MyDogDNA™ assay,^2^ which includes both a canine within-individual heterozygosity test, and multiplex DNA panel screening for known inherited genetic disease variants (Additional file 1: Table S1) and traits such as coat varieties. The inclusion of the panel screening offers the opportunity to explore possible predispositions or exclude known disease variants in the breeding strategy.

The aim of this paper is to evaluate the measurement of genetic diversity and multiplex DNA panel screening for implementation in a breeding strategy for the Dutch Shepherd Dog (DSD).

The Von Willebrand’s Disease type I (VWD-I) gene mutation c.7437G > A (p.Ser2479Ser, OMIA ref: [24]) was identified in a single long haired DSD during this study, a mutation that has so far been found in at least 20 breeds or breed variants [25]. Thus, assessing the prevalence of the VWD-I mutation and the clinical consequences in the DSD population emerged as a second aim.

## Results

**Fig. 1:**
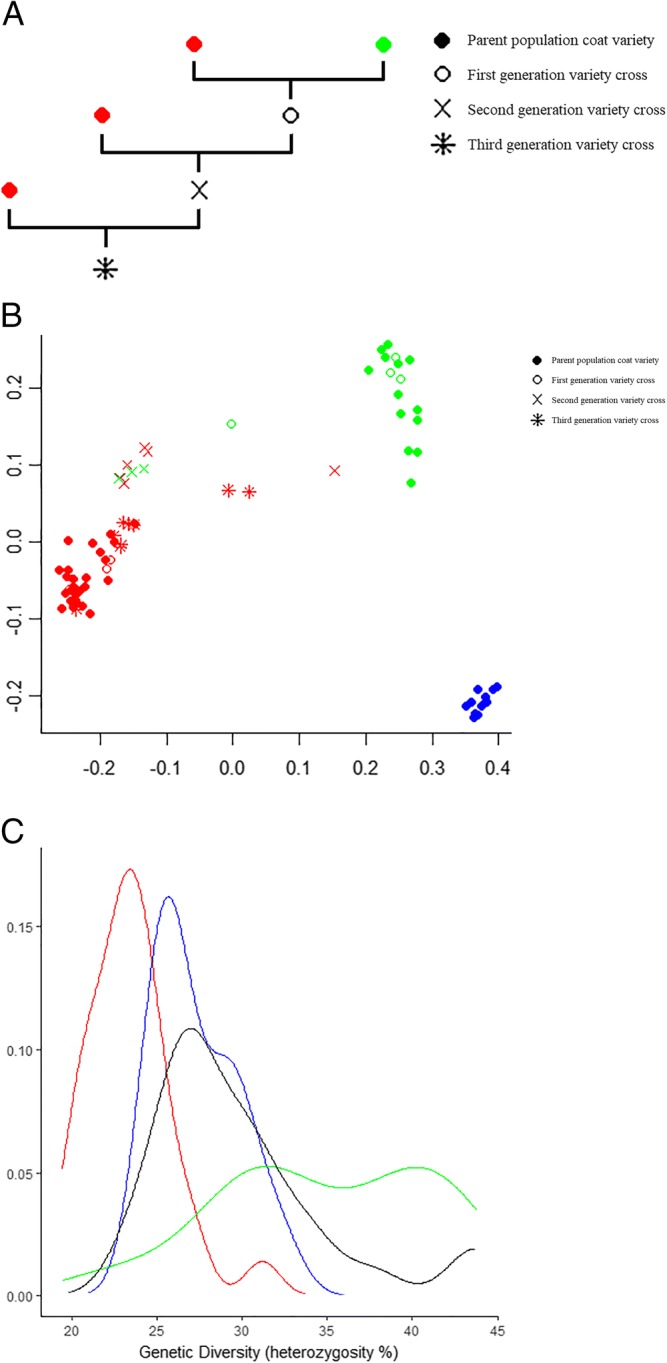
Genetic relationship and diversity in the Dutch Shepherd Dog. **a** Crossbreeding of short haired and long haired Dutch Shepherd Dogs. Parent populations of short haired (green) and long haired (red) dogs were matched to produce first generation variety crosses. Matching variety crosses with a parent population (backcross) resulted in the next generation of variety crosses. **b** Genetic relationship of the tested population of Dutch Shepherd Dogs in a multidimensional scaling plot. The parent populations shown are short haired (green, *n* = 13), long haired (red, *n* = 28), and wire haired (blue, *n* = 13) varieties. Variety crosses are shown in the colour of the genetically confirmed coat variety (trait testing MyDogDNA™). **c** Genetic diversity of the tested population of Dutch Shepherd Dogs. The short haired (green, *n* = 18), long haired (red, *n* = 46), and wire haired (blue, *n* = 16) are shown together with a combination of the variety crosses between long and short haired (black, *n* = 25)

Figure 1a shows the method of crossbreeding and backcrossing coat varieties between short haired and long haired DSDs, resulting in several types of variety crosses.

### MyDogDNA™ testing

The genetic relationships are shown through a multidimensional scaling plot (Fig. 1b). The visual representation of genetic relationships shows the separation between the coat varieties of the DSDs. The progeny resulting from crossbreeding between the short haired and long haired coat varieties are included in the plot as variety crosses and shown in the colour of the genetically confirmed coat variety (trait testing MyDogDNA™). The coat genotype and phenotype agree in all cases.

The median genetic diversity of the short haired DSDs was significantly higher than that of the other two varieties (38.3% for the short haired DSD versus 25.4 and 26.7% for the long haired and the wire haired respectively (*p* < 0.05)). All variety crosses of short haired x long haired together had a genetic diversity of 29.4%, which was significantly higher than the 25.4% of the long haired parent population (*p* < 0.05) (Fig. 1c).

**Fig. 2:**
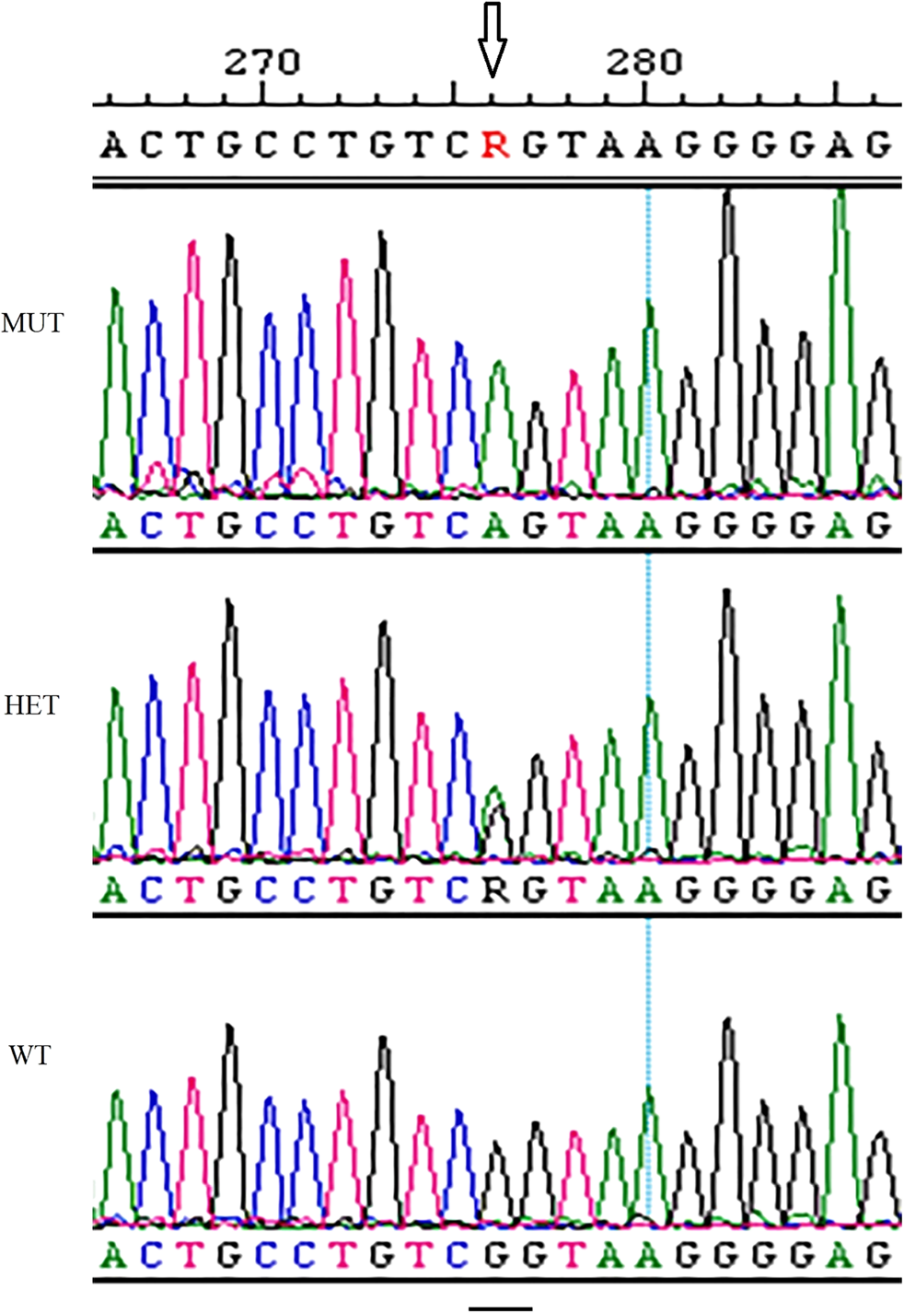
Von Willebrand’s Factor mutation analysis. Example of chromosomal DNA containing Von Willebrand’s Factor c.7437G > A. WT = wildtype, HET = heterozygous carrier, MUT = homozygous mutant

**Fig. 3:**
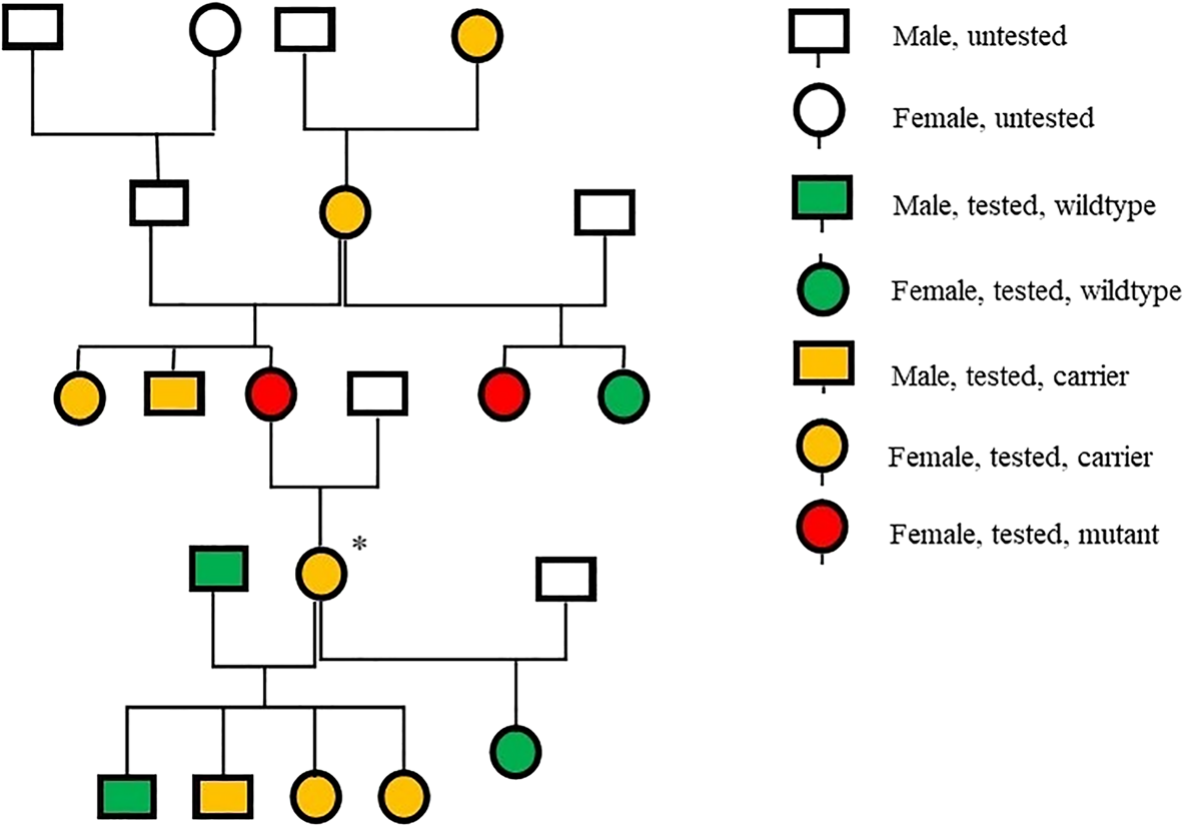
Von Willebrand’s Disease segregation in the Dutch Shepherd Dog. Dutch Shepherd Dog pedigree following first identification (*) of a carrier of Von Willebrand’s Disease type I. This individual was a female long haired shepherd born in 2010.

Results of the disease variant panel screening in the 30 DSDs that were tested in the first phase of this study showed one carrier for VWD-I in the long haired DSD variety, in which a c.7437G > A variant was present (Fig. 2). All dogs were clear for the remaining 188 disease-causing mutations present on the MyDogDNA™ array. A structural or quantitative defect in the Von Willebrand’s Factor (VWF) [26] leads to a bleeding disorder called Von Willebrand’s Disease (VWD) [27]. VWD-I is characterized by a decrease in the concentration of plasma VWF. VWD-I is associated with mild clinical signs only [27]. To assess whether the VWD-I mutation was a de novo mutation or a segregating mutation, family members of the VWD-I carrier were subsequently tested with MyDogDNA™, which identified multiple carriers, as well as two homozygous individuals (Fig. 3), indicating that it was a segregating mutation.

### Allele frequency VWD-I gene in population

Combining the results of the panel screening with the Sanger sequencing, the cross-section of the long haired breeding population from 2013 to 2015 (*n* = 42, 89% of the dogs from the Dutch population used for breeding) showed an allele frequency of < 3% (2 alleles of 84 tested alleles), since two breeding individuals were carriers and no homozygously affected individuals were found (Additional file 1: Figure S1).

### Evaluation of the bleeding history and coagulation

**Fig. 4:**
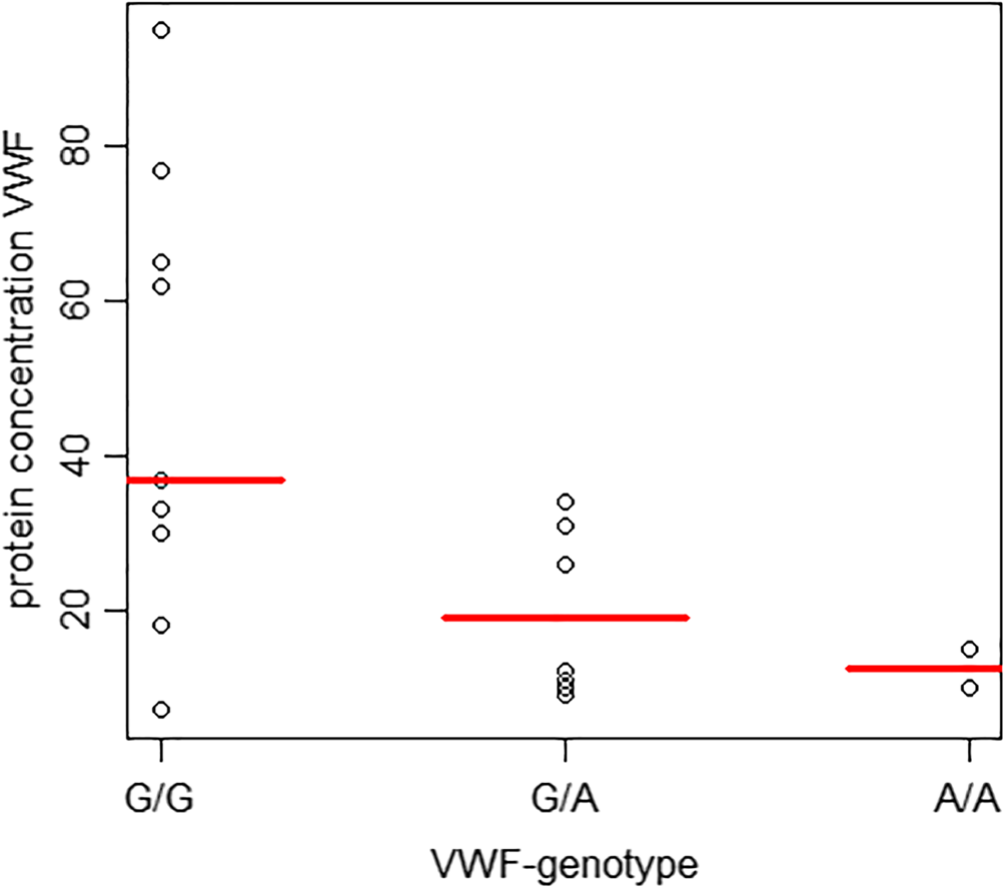
Von Willebrand’s Factor protein concentration and genotype correlation in the Dutch Shepherd Dog. Correlation between Von Willebrand’s Factor protein concentrations in blood (median shown in red) and Von Willebrand’s Disease genotype (G/G = wildtype (*n* = 9), G/A = heterozygous carrier (*n* = 8), A/A = homozygous mutant (*n* = 2)), in 19 Dutch Shepherd Dogs. No significant difference in protein concentration was found when the three groups were compared (Kruskal-Wallis test, *p* = 0.07). Comparing the wildtype group with the other two groups combined showed a significant difference in protein concentration (Mann-Whitney U test, *p* = 0.03)

Eight out of 19 owners of the dogs included in the clinical validation experiment reported that their dog had experienced a bleeding episode (genotypes in these eight dogs were wildtype (4), heterozygous carrier (2), and homozygous mutant (2)). However, all of these episodes could be related to trauma, no excessive bleeding was reported. VWF protein concentrations ranged between 7 and 95%. No significant difference in VWF values was found when the three groups were compared (Kruskal-Wallis test, *p* = 0.07). We found a significant difference in the Von Willebrand protein concentration when comparing the wildtype group with the other two groups combined (Mann-Whitney U test, *p* = 0.03) (Fig. 4 and Table 1). PT, aPTT, and fibrinogen were within reference range in all 19 dogs. Thrombocytes were below reference range in one dog, which was thought to be related to thrombocyte aggregates identified in the blood smear.

**Table 1:** Individual determination of genotype and coagulation profile in the Dutch Shepherd Dog

Dog #	Genotype	VWF	Thrombocytes	Fibrinogen
1	G/G	7	344	1.6
2	G/G	18	257	1.6
3	G/G	30	344	1.8
4	G/G	33	322	1.8
5	G/G	37	97*	4.2*
6	G/G	62	342	1.4
7	G/G	65	338	1.8
8	G/G	77	358	1.6
9	G/G	95	395	3.2
10	G/A	9	284	1.8
11	G/A	10	305	1.7
12	G/A	11	366	2
13	G/A	12	381	2.1
14	G/A	26	247	1.1
15	G/A	31	330	2
16	G/A	31	334	1.9
17	G/A	34	360	1.9
18	A/A	10	321	3.7
19	A/A	15	303	1.6

Results for Von Willebrand’s Factor protein concentrations in blood, Von Willebrand’s Disease genotype (G/G = wildtype, G/A = heterozygous carrier, A/A = homozygous mutant), thrombocytes (ref. 144–603 10^9^/L) and fibrinogen (ref. 1.0–2.7 g/L) in 19 Dutch Shepherd Dogs. *Many thrombocyte aggregates present

## Discussion

The aim of this paper was to illustrate how heterozygosity testing through genome-wide SNP testing combined with multiplex DNA panel screening could be applied in sensible breeding advice. In the current study, the MyDogDNA™ assay was used because of logistics, as well as the fact that the DSD breeders had already sent samples there to assess genetic diversity of their breed. Other institutes providing similar genetic diversity testing include the University of California^3^ and the University of Cornell in New York^4^ [28, 29]. The MyDogDNA™ assay was deemed a valid tool, as after an extensive validation and development phase on approximately 7000 dogs representing over 230 breeds, the panel screening was shown to be instrumental in the detection of causative mutations that were previously undocumented in certain breeds [30, 31], as was the case in the DSD. However, the absence of mutations does not necessarily equate to the absence of the disease allele or clinical disease, since different mutations in different dog breeds may lead to the same clinical disease. Since unidentified disease mutations may also be present, continued expansion of test panel content is paramount [30]. Breeding for certain qualities and health is a multifaceted issue. Donner et al. previously discussed the applicability of the tool and its place as part of a holistic breeding strategy [30].

Individual test results should not in themselves lead to exclusion from breeding without knowledge of the pathophysiology of the disease and the connected test result. Careful interpretation of results and validation in the new population should be part of a breeding strategy including multiple tools [12].

The genetic relationships plot shows a distance between the DSD coat varieties, suggesting genetic isolation occurs not only between breeds in general, but also between subgroups of a breed if isolated populations are created. It can be seen that crossbreeding between DSD varieties bridges this genetic isolation. Allowing further crossbreeding may therefore increase the potential for choosing the best genetic diversity-increasing match within the DSD population, while conserving desired coat varieties as breed-specific trait.

The genetic diversity of two of the three DSD coat varieties is less than the median diversity of all combined purebred dogs (33.8%). The short haired DSDs have a greater level of diversity. So, in relation to purebred dogs as a whole, our observations showed that the DSD is at the lower end of the spectrum. The genetic diversity of all three DSD varieties is less than that of mixed-breed dogs (43.2%) [32]. Being aware of the variation in mixed-breed dogs, this last result could be expected. The short haired DSD population has the highest diversity which is most likely due to the fact that the effective population size is larger compared to the other two coat varieties. It may not, therefore, be in immediate need of crossbreeding to maintain a healthy gene pool, but it may be used to increase the diversity within the other two coat varieties. As most testing was done throughout the breadth of the DSD gene pool, we consider the genetic diversity measurements to be a fair representation of the true genetic diversity.

Breeding for heterozygosity reduces the risk of inbreeding depression, where accumulation of deleterious mutations leads to a lower individual fitness. This may lead to smaller litters, reduced lifespan and increased mortality in offspring [33]. Although individual benefits are not yet apparent, breeding for heterozygosity aims at maintaining the population gene pool [12]. In this study, the aim was to explore which insight on the DSD breed was provided by genetic diversity analysis. We identified an increased homozygosity within the three subpopulations of coat varieties which were previously not allowed to breed with each other. Although no obvious health issues were reported until now, continuous breeding within the subgroups and selection will likely lead to more loss of genetic diversity and carries a risk of future negative influence of recessive alleles.

We would in this case advise expanding the effective population size for each coat variety; to make full use of the available gene pool whilst selecting animals with the desired characteristics for the breed. To increase the heterozygosity within the three different coat varieties, we advise to continue variety crossbreeding. It is important to note that the DSD remains a distinct dog breed in this way, but the separation between the coat varieties will decrease, decreasing the risk of accumulation of recessive alleles within coat varieties. Even with breeding between the coat varieties, one of the important desired breed characteristics (coat-length) for the DSD was maintained and future selection of dogs for breeding could be supported by using the tests for traits that are present on MyDogDNA™.

The results of the VWD-I sequencing show an allele frequency of < 3% in the DSD breeding population in the years 2013–2015 in the Netherlands. This breeding population is assumed to be the parent population of the current national DSD population. In the subset of 19 dogs that were clinically evaluated, no bleeding tendency was found, although we observed a statistically significant lower VWF protein concentration in dogs hetero- or homozygous for the examined VWD-I mutation. The results of this limited sample confirm that, as in other breeds with VWD-I, the presence of a mutated allele leads to a lower VWD protein concentration but shows only limited signs of haemorrhagic diathesis [34–36]. However, the predictive value of common coagulation tests may be limited [37].This underlines the importance of assessing the phenotype associated with the mutation. Although VWD-I disease usually gives mild clinical phenotype, when additional trauma is present, the disease could lead to clinically relevant bleeding. Therefore we advise to prevent homozygous mutants arising from breeding. In the DSD population, this should be feasible without excluding breeding animals from the population, as the VWD-I allele frequency within the DSD is low.

## Conclusions

Increased inbreeding of (sub) populations of a dog breed, carries the risk of inbreeding depression and increase of allele frequency of disease-causing, usually recessive alleles. Increasing heterozygosity, whilst maintaining characteristics important for the breed, and prevention the segregation of disease-causing gene mutations may be important in a sustainable, healthy breeding program.

Genetic relationship measurements can be used to match breeding couples to increase the genetic diversity in a breed population or in subpopulations within a breed. The multiplex DNA panel screening can be used to check for genetic disorders in the breed that were previously unknown and could potentially spread unintendedly in the population.

A sensible breeding programme should include application of the described genetic tools with appropriate counselling, as well as individual and population-based clinical screening for disorders with and without a known mutation.

## Material & methods

### Dogs

Members of the Dutch Shepherd Dog Club volunteered the individual DSDs tested in this study. The numbers in each of the consecutive steps were 1) MyDogDNA™ screening first testing group (10 short haired, 10 long haired, 10 wire haired); 2) Von Willebrand’s Factor (VWF) type I genotype testing in the long haired DSD population through continued MyDogDNA™ testing and Sanger sequencing (14 in pedigree first identified individual, 42 long haired Dutch breeding population 2013–2015); 3) MyDogDNA™ combined results (13 short haired, 28 long haired, and 13 wire haired for the genetic relationships; 18 short haired, 46 long haired, 16 wire haired, and 25 variety crosses for the heterozygosity); and 4) Evaluation of the bleeding history and coagulation (19 individuals, of which nine wildtype, eight carriers and two homozygously affected).

Crossbreeding of coat varieties in this study took place between short haired and long haired DSDs only. Any crossbreeding between the short haired and wire haired variety is not included in this study, and crossbreeding between long haired and wire haired varieties is not allowed. The parent population of short haired and long haired DSDs were matched (in effect, mated) to produce first generation variety crosses. Matching first generation variety crosses with a parent population (backcross) resulted in second generation variety crosses, matching second generation variety crosses with a parent population resulted in third generation variety crosses (Fig. 1a).

### MyDogDNA™ testing

MyDogDNA™ (Genoscoper Laboratories Oy, Helsinki, Finland) testing consists of two main tests: heterozygosity testing, and multiplex DNA panel screening. Heterozygosity is determined using a genome-wide single nucleotide polymorphism (SNP) test – evaluating genetic relationships and genetic diversity, respectively relating to the individual and the population (e.g. [38]). For the present study, MyDogDNA™ derived genotypes were available for 2642 SNPs. Genetic diversity is expressed as SNP heterozygosity ratio, in effect the proportion of heterozygous SNPs out of all examined SNPs. The statistical testing of the median genetic diversity was carried out non-parametrically with a Kruskal-Wallis test (*p* < 0.05) The multiplex DNA panel screening is a genotyping microarray, which, at the time of the present study, tested for 189 known disease variants and 22 traits, including coat length and colour [32] (Additional file 1: Table S1). The validation and power of the panel as a research discovery tool was previously described in detail by Donner et al. [30, 31].

### Sequencing of the VWD-I gene

Sanger sequencing was performed as follows. DNA was isolated from Oragene Animal-400 saliva swabs using the manufacturer’s instructions (DNA genotek). We performed PCR to capture the VWD-I mutation (forward primer (5′- AAATCTCCTTCATAAGCATCCC-3′) and reverse primer (5′- CTGCCTTTCACCCAACCT-3′)). The PCR product was treated with Exonuclease I and Shrimp Alkaline Phosphatase. Sequence reactions, performed with Big Dye Terminator Ready Reaction Mix v3.1 (Applied Biosystems), were sequenced on an ABI3500XL and analyzed in Lasergene (version 12.0 DNASTAR).

### Evaluation of the bleeding history and coagulation

To evaluate the clinical significance of the VWD-I mutation, haemostasis was assessed in 19 DSDs in the following numbers - wildtype (*n* = 9), carriers (*n* = 8) or homozygously affected (*n* = 2). A detailed history, VWF concentration, coagulation profile, and thrombocyte count were collected. Blood samples from the jugular vein (4 ml sodium citrate 3.8%, 4 ml EDTA) were used to determine the VWF I antigen concentration by enzyme-linked immunosorbent assay (ELISA [39],), coagulation parameters (Prothrombin Time (PT), activated Partial Thromboplastin Time (aPTT), fibrinogen), and thrombocyte count. All tests were performed in the University Veterinary Diagnostic Laboratory (Utrecht University). The difference in VWF distribution between the three genetic groups were tested non-parametrically using the Kruskal-Wallis test. The difference between the wildtype group and the combined group of heterozygous and homozygous individuals was tested non-parametrically using the Mann-Whitney U test. Significance level was set at *p* < 0.05 for both tests.

## Additional file

Additional file 1: **Table S1.** Multiplex DNA panel screening. Tested disorders within the multiplex DNA panel screening by MyDogDNA™, organised by type of disorder. **Figure S1.** Cross-section 2013–2015 Dutch breeding population of the long haired Dutch Shepherd Dog. Cross-section of the long haired Dutch Shepherd Dog breeding population for the years 2013–2015 in the Netherlands, combining the results of MyDogDNA™ multiplex DNA panel screening with Sanger sequencing for the causal variant for Von Willebrand’s Disease type I (89%, *n* = 42 from 47 breeding individuals). Litters are shown by birth year, most were not related. Resulting allele frequency in the breeding population is 2%. (DOCX 186 kb)

## Abbreviations

DSD: Dutch Shepherd Dog
SNP: Single nucleotide polymorphism
VWD-I: Von Willebrand’s Disease type I
VWF: Von Willebrand’s Factor
SNP: single nucleotide polymorphism
DSD: Dutch Shepherd Dog

## Footnotes

1 See: http://www.hollandseherder.nl/details/the_dutch_shepherd/.

2 See: http://www.mydogdna.com.

3 See: https://www.vgl.ucdavis.edu.

4 See: https://embarkvet.com.
