# Supplementary material for: Heterozygosity testing and multiplex DNA panel screening as a potential tool to monitor health and inbreeding in a small, closed dog population
Source: Canine Genet Epidemiol. 2018 Dec 28;5:12. doi: 10.1186/s40575-018-0068-6 (PMC6309085; doi:10.1186/s40575-018-0068-6)
Supplement: Supplementary file 1 — Table S1. Multiplex DNA panel screening. Tested disorders within the multiplex DNA panel screening by MyDogDNA™, organised by type of disorder. Figure S1. Cross-section 2013–2015 Dutch breeding population of the long haired Dutch Shepherd Dog. Cross-section of the long haired Dutch Shepherd Dog breeding population for the years 2013–2015 in the Netherlands, combining the results of MyDogDNA™ multiplex DNA panel screening with Sanger sequencing for the causal variant for Von Willebrand’s Disease type I (89%, n = 42 from 47 breeding individuals). Litters are shown by birth year, most were not related. Resulting allele frequency in the breeding population is 2%. (DOCX 186 kb) [file 40575_2018_68_MOESM1_ESM.docx]

**Supplemental information, Figure 1: Cross-section 2013-2015 Dutch breeding population of the long haired Dutch Shepherd Dog**

Cross-section of the long haired Dutch Shepherd Dog breeding population for the years 2013-2015 in the Netherlands, combining the results of MyDogDNA™ multiplex DNA panel screening with Sanger sequencing for the causal variant for Von Willebrand’s Disease type I (89%, n=42 from 47 breeding individuals). Litters are shown by birth year, most were not related. Resulting allele frequency in the breeding population is 2%.


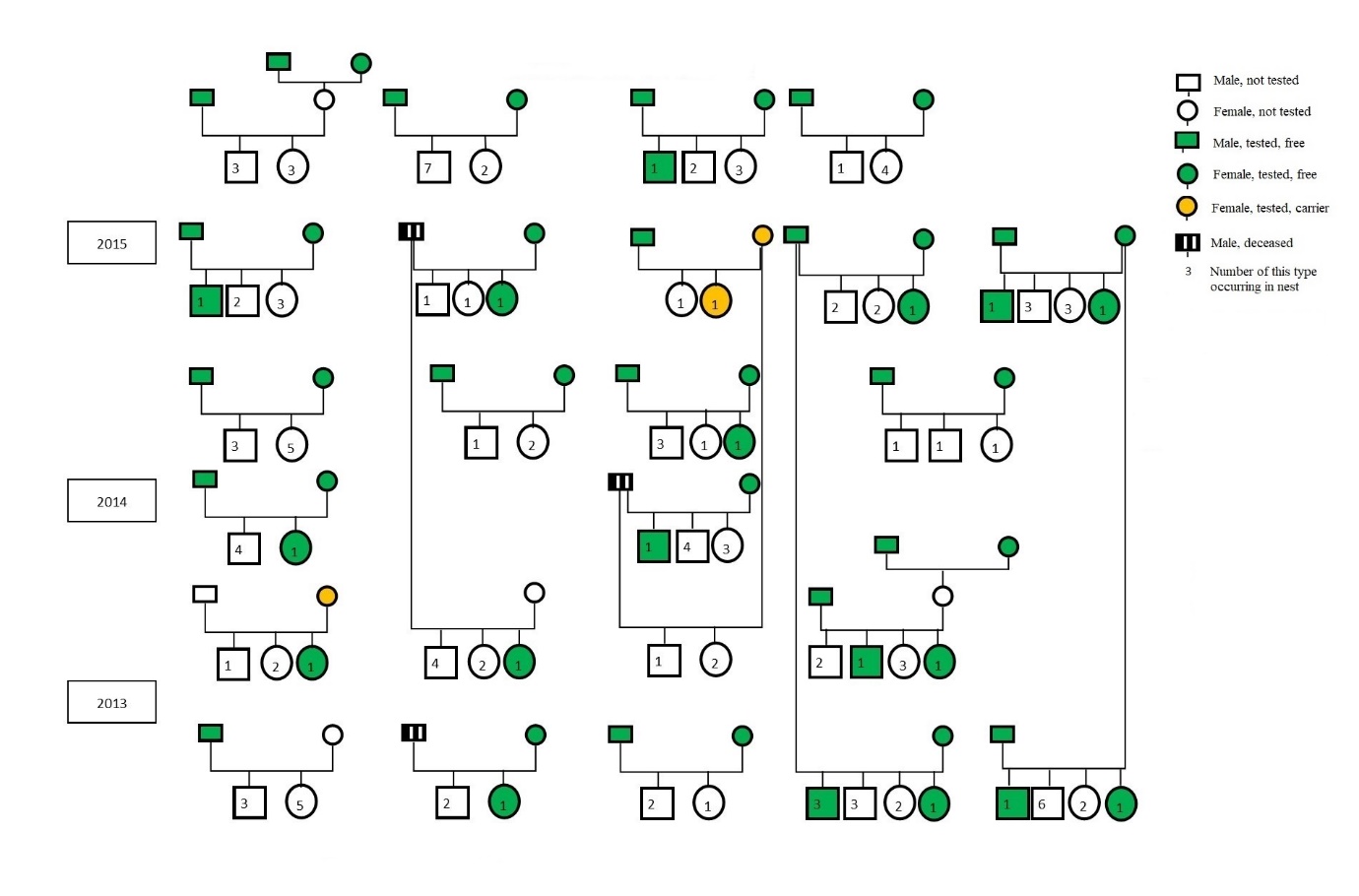


**Supplemental information, Table 1: Multiplex DNA panel screening**

Tested disorders within the multiplex DNA panel screening by MyDogDNA™, organised by type of disorder.

| *Type of disorder* | *Disorder* |
| --- | --- |
| Blood | Bleeding disorder due to P2RY12 defect |
| Blood | Canine Cyclic Neutropenia, Cyclic Hematopoiesis, Grey Collie Syndrome, (CN) |
| Blood | Canine Leukocyte Adhesion Deficiency (CLAD), type III |
| Blood | Canine Scott Syndrome, (CSS) |
| Blood | Factor IX Deficiency or Haemophilia B; mutation Gly379Glu |
| Blood | Factor IX Deficiency or Haemophilia B; mutation originally found in Airedale Terrier |
| Blood | Factor IX Deficiency or Haemophilia B; mutation originally found in German Wirehaired Pointer |
| Blood | Factor IX Deficiency or Haemophilia B; mutation originally found in Lhasa Apso |
| Blood | Factor VII Deficiency |
| Blood | Factor VIII Deficiency or Haemophilia A; mutation originally found in Boxer |
| Blood | Factor VIII Deficiency or Haemophilia A; mutation originally found in German Shepherd Dog |
| Blood | Factor VIII Deficiency or Haemophilia A; mutation originally found in Old English Sheepdog |
| Blood | Factor VIII Deficiency or Haemophilia A; p.Cys548Tyr mutation originally found in German Shepherd |
| Blood | Factor XI Deficiency |
| Blood | Glanzmann Thrombasthenia Type I, (GT); mutation originally found in mixed-breed dogs |
| Blood | Glanzmann Thrombasthenia Type I, (GT); mutation originally found in Pyrenean Mountain Dog |
| Blood | Hereditary Elliptocytosis |
| Blood | Hereditary Phosphofructokinase (PFK) Deficiency |
| Blood | Macrothrombocytopenia; disease-linked variant originally found in Norfolk and Cairn Terrier |
| Blood | May-Hegglin Anomaly (MHA) |
| Blood | Prekallikrein Deficiency |
| Blood | Pyruvate Kinase Deficiency; mutation originally found in Basenji |
| Blood | Pyruvate Kinase Deficiency; mutation originally found in Beagle |
| Blood | Pyruvate Kinase Deficiency; mutation originally found in Pug |
| Blood | Pyruvate Kinase Deficiency; mutation originally found in West Highland White Terrier |
| Blood | Thrombopathia; mutation originally found in Basset Hound |
| Blood | Thrombopathia; mutation originally found in Eskimo Spitz |
| Blood | Thrombopathia; mutation originally found in Landseer |
| Blood | Trapped Neutrophil Syndrome, (TNS) |
| Blood | Von Willebrand's Disease (vWD) Type I |
| Blood | Von Willebrand's Disease (vWD) Type III; mutation originally found in Kooikerhondje |
| Blood | Von Willebrand's Disease (vWD) Type III; mutation originally found in Scottish Terrier |
| Blood | Von Willebrand's Disease (vWD) Type III; mutation originally found in Shetland Sheepdog |
| Cardiac | Dilated Cardiomyopathy, (DCM); mutation originally found in Schnauzer |
| Cardiac | Long QT Syndrome |
| Dermal | Dystrophic Epidermolysis Bullosa; mutation originally found in Central Asian Ovcharka |
| Dermal | Dystrophic Epidermolysis Bullosa; mutation originally found in Golden Retriever |
| Dermal | Epidermolytic Hyperkeratosis |
| Dermal | Focal Non-Epidermolytic Palmoplantar Keratoderma, (FNEPPK); mutation originally found in Dogue de Bordeaux |
| Dermal | Hereditary Footpad Hyperkeratosis, (HFH) |
| Dermal | Ichthyosis; mutation originally found in American Bulldog |
| Dermal | Ichthyosis; mutation originally found in Great Dane |
| Dermal | Lamellar Ichthyosis, (LI) |
| Dermal | Ligneous Membranitis |
| Dermal | Musladin-Lueke syndrome, (MLS) |
| Dermal | X-Linked Ectodermal Dysplasia, (XHED) |
| Endocrine Disorders | Congenital Hypothyroidism; mutation originally found in Tenterfield Terrier |
| Endocrine Disorders | Congenital Hypothyroidism; mutation originally found in Toy Fox and Rat Terrier |
| Immunological Disorders | Autosomal Recessive Severe Combined Immunodeficiency, (ARSCID) |
| Immunological Disorders | Complement 3 (C3) Deficiency |
| Immunological Disorders | Myeloperoxidase Deficiency |
| Immunological Disorders | Severe Combined Immunodeficiency in Frisian Water Dogs, (SCID) |
| Immunological Disorders | X-Linked Severe Combined Immunodeficiency (XSCID); mutation originally found in Basset Hound |
| Immunological Disorders | X-Linked Severe Combined Immunodeficiency (XSCID); mutation originally found in Cardigan Welsh Corgi |
| Metabolic Disorders | Glycogen Storage Disease Type Ia, (GSD Ia) |
| Metabolic Disorders | Glycogen Storage Disease Type II or Pompe's Disease, (GSD II) |
| Metabolic Disorders | Glycogen Storage Disease Type IIIa, (GSD IIIa) |
| Metabolic Disorders | Hypocatalasia or Acatalasemia |
| Metabolic Disorders | Intestinal Cobalamin Malabsorption or Imerslund-Gräsbeck Syndrome, (IGS); mutation originally found in Beagle |
| Metabolic Disorders | Intestinal Cobalamin Malabsorption or Imerslund-Gräsbeck Syndrome, (IGS); mutation originally found in Border Collie |
| Metabolic Disorders | Mucopolysaccharidosis Type IIIA, (MPS IIIA); mutation originally found in Dachshund |
| Metabolic Disorders | Mucopolysaccharidosis Type IIIA, (MPS IIIA); mutation originally found in New Zealand Huntaway |
| Metabolic Disorders | Mucopolysaccharidosis Type VII, (MPS VII); mutation originally found in Brazilian Terrier |
| Metabolic Disorders | Mucopolysaccharidosis Type VII, (MPS VII); mutation originally found in German Shepherd |
| Metabolic Disorders | Pyruvate Dehydrogenase Phosphatase 1 (PDP1) Deficiency |
| Muscular Disorders | Cavalier King Charles Spaniel Muscular Dystrophy, (CKCS-MD) |
| Muscular Disorders | Centronuclear Myopathy, (CNM); mutation originally found in Great Dane |
| Muscular Disorders | Centronuclear Myopathy, (CNM); mutation originally found in Labrador Retriever |
| Muscular Disorders | Duchenne or Dystrophin Muscular Dystrophy, (DMD); mutation originally found in Golden Retriever |
| Muscular Disorders | Duchenne or Dystrophin Muscular Dystrophy, (DMD); mutation originally found in Norfolk Terrier |
| Muscular Disorders | Muscular Dystrophy, Ullrich-type; mutation originally found in Landseer |
| Muscular Disorders | Myostatin deficiency (Double Muscling, "Bully") |
| Muscular Disorders | Myotonia Congenita; mutation originally found in Australian Cattle Dog |
| Muscular Disorders | Myotonia Congenita; mutation originally found in Miniature Schnauzer |
| Muscular Disorders | Myotubular Myopathy; mutation originally found in Rottweiler |
| Muscular Disorders | Nemaline Myopathy; mutation originally found in American Bulldog |
| Muscular Disorders | X-Linked Myotubular Myopathy |
| Neurological Disorders | Acral Mutilation Syndrome, (AMS) |
| Neurological Disorders | Alaskan Husky Encephalopathy, (AHE) |
| Neurological Disorders | Alexander Disease (AxD); mutation originally found in Labrador Retriever |
| Neurological Disorders | Bandera's Neonatal Ataxia, (BNAt) |
| Neurological Disorders | Benign Familial Juvenile Epilepsy or Remitting Focal Epilepsy |
| Neurological Disorders | Cerebellar Cortical Degeneration, (CCD); mutation originally found in Vizsla |
| Neurological Disorders | Cerebral Dysfunction; mutation originally found in Friesian Stabyhoun |
| Neurological Disorders | Dandy-Walker-Like Malformation (DWLM); mutation originally found in Eurasier |
| Neurological Disorders | Early-Onset Progressive Polyneuropathy; mutation originally found in Alaskan Malamute |
| Neurological Disorders | Fetal Onset Neuroaxonal Dystrophy, (FNAD) |
| Neurological Disorders | Hereditary Ataxia or Cerebellar Ataxia; mutation originally found in Old English Sheepdog and Gordon Setter |
| Neurological Disorders | Hyperekplexia or Startle Disease |
| Neurological Disorders | Hypomyelination; mutation originally found in Weimaraner |
| Neurological Disorders | Juvenile Myoclonic Epilepsy, (JME); mutation originally found in Rhodesian Ridgeback |
| Neurological Disorders | L-2-Hydroxyglutaric aciduria, (L2HGA); mutation originally found in Staffordshire Bull Terrier |
| Neurological Disorders | L-2-Hydroxyglutaric aciduria, (L2HGA); mutation originally found in West Highland White Terrier |
| Neurological Disorders | Lagotto Storage Disease, (LSD) |
| Neurological Disorders | Neonatal Cerebellar Cortical Degeneration or Cerebellar Abiotrophy, (NCCD) |
| Neurological Disorders | Neonatal Encephalopathy with Seizures, (NEWS) |
| Neurological Disorders | Neuroaxonal Dystrophy (NAD); mutation originally found in Spanish Water Dog |
| Neurological Disorders | Neuronal Ceroid Lipofuscinosis 1, (NCL1); mutation originally found in Dachshund |
| Neurological Disorders | Neuronal Ceroid Lipofuscinosis 10, (NCL10); mutation originally found in American Bulldog |
| Neurological Disorders | Neuronal Ceroid Lipofuscinosis 5, (NCL5); mutation originally found in Border Collie |
| Neurological Disorders | Neuronal Ceroid Lipofuscinosis 8, (NCL8); mutation originally found in Alpine Dachsbracke |
| Neurological Disorders | Neuronal Ceroid Lipofuscinosis 8, (NCL8); mutation originally found in Australian Shepherd |
| Neurological Disorders | Neuronal Ceroid Lipofuscinosis 8, (NCL8); mutation originally found in English Setter |
| Neurological Disorders | Neuronal Ceroid Lipofuscinosis, (NCL7); mutation originally found in Chinese Crested Dog and Chihuahua |
| Neurological Disorders | Polyneuropathy with ocular abnormalities and neuronal vacuolation, (POANV); mutation originally found in Black Russian Terrier |
| Neurological Disorders | Progressive Early-Onset Cerebellar Ataxia; mutation originally found in Finnish Hound |
| Neurological Disorders | Sensory Neuropathy; mutation originally found in Border Collie |
| Neurological Disorders | Spinal Dysraphism |
| Neurological Disorders | Spinocerebellar Ataxia with Myokymia and/or Seizures (SCA) |
| Neurological Disorders | Spinocerebellar Ataxia/ Late-Onset Ataxia (SCA, LOA) |
| Neurological Disorders | Spongy degeneration with cerebellar ataxia, (SDCA1); mutation originally found in Belgian Shepherd Dog |
| Neurological Disorders | X-Linked Tremors; mutation originally found in English Springer Spaniel |
| Neuromuscular Disorders | Congenital Myasthenic Syndrome (CMS); mutation originally found in Labrador Retriever |
| Neuromuscular Disorders | Congenital Myasthenic Syndrome, (CMS); mutation originally found in Jack Russell Terrier |
| Neuromuscular Disorders | Congenital Myasthenic Syndrome, (CMS); mutation originally found in Old Danish Pointing Dog |
| Neuromuscular Disorders | Globoid Cell Leukodystrophy or Krabbe Disease, (GLD); mutation originally found in Irish Setter |
| Neuromuscular Disorders | Globoid Cell Leukodystrophy or Krabbe Disease, (GLD); mutation originally found in Terriers |
| Neuromuscular Disorders | GM1 Gangliosidosis; mutation originally found in Alaskan Husky |
| Neuromuscular Disorders | GM1 Gangliosidosis; mutation originally found in Portuguese Water Dog |
| Neuromuscular Disorders | GM1 Gangliosidosis; mutation originally found in Shiba Dog |
| Neuromuscular Disorders | GM2 Gangliosidosis, mutation originally found in Japanese Chin |
| Neuromuscular Disorders | GM2 Gangliosidosis; mutation originally found in Toy Poodle |
| Neuromuscular Disorders | Paroxysmal Dyskinesia, (PxD); mutation originally found in Irish Soft Coated Wheaten Terrier |
| Ocular | Canine Multifocal Retinopathy 1, (CMR1); mutation originally found in Mastiff-related breeds |
| Ocular | Canine Multifocal Retinopathy 2, (CMR2); mutation originally found in Coton de Tulear |
| Ocular | Canine Multifocal Retinopathy 3, (CMR3); mutation originally found in Lapponian Herder |
| Ocular | Cone Degeneration, (CD) or Achromatopsia; mutation originally found in Alaskan Malamute |
| Ocular | Cone Degeneration, (CD) or Achromatopsia; mutation originally found in German Shepherd Dog |
| Ocular | Cone Degeneration, (CD) or Achromatopsia; mutation originally found in German Shorthaired Pointer |
| Ocular | Cone-Rod Dystrophy 1, (crd1); mutation originally found in American Staffordshire Terrier |
| Ocular | Cone-Rod Dystrophy 2, (crd2); mutation originally found in American Pit Bull Terrier |
| Ocular | Cone-Rod Dystrophy, (cord1-PRA / crd4) |
| Ocular | Cone-Rod Dystrophy, Standard Wirehaired Dachshund, (crd SWD) |
| Ocular | Congenital Stationary Night Blindness (CSNB) |
| Ocular | Dominant Progressive Retinal Atrophy, (DPRA) |
| Ocular | Generalised Progressive Retinal Atrophy |
| Ocular | Golden Retriever Progressive Retinal Atrophy 1, (GR_PRA 1) |
| Ocular | Primary Hereditary Cataract, (PHC); mutation originally found in Australian Shepherd |
| Ocular | Primary lens luxation (PLL) and glaucoma; mutation originally found in Shar Pei |
| Ocular | Primary Lens Luxation, (PLL) |
| Ocular | Primary Open Angle Glaucoma, (POAG); mutation originally found in Basset Fauve de Bretagne |
| Ocular | Primary Open Angle Glaucoma, (POAG); mutation originally found in Beagle |
| Ocular | Primary Open Angle Glaucoma, (POAG); mutation originally found in Norwegian Elkhound |
| Ocular | Primary Open Angle Glaucoma, (POAG); mutation originally found in Petit Basset Griffon Vendeen |
| Ocular | Progressive Retinal Atrophy Type III, (PRA type III); mutation originally found in Tibetan Spaniel and Tibetan Terrier |
| Ocular | Progressive Retinal Atrophy, (CNGA1-PRA); mutation originally found in Shetland Sheepdog |
| Ocular | Progressive Retinal Atrophy, (PAP1_PRA); mutation originally found in Papillon and Phalene |
| Ocular | Progressive Retinal Atrophy, (PRA); mutation originally found in Basenji |
| Ocular | Progressive Retinal Atrophy, (PRA); mutation originally found in Swedish Vallhund |
| Ocular | Rod-Cone Dysplasia 1, (rcd1); mutation originally found in Irish Setter |
| Ocular | Rod-Cone Dysplasia 1a, (rdc1a); mutation originally found in Sloughi |
| Ocular | Rod-Cone Dysplasia 3, (rcd3) |
| Ocular | X-Linked Progressive Retinal Atrophy 1, (XLPRA1) |
| Ocular | X-Linked Progressive Retinal Atrophy 2, (XLPRA2) |
| Other | Acute Respiratory Distress Syndrome, (ARDS); mutation originally found in Dalmatian |
| Other | Amelogenesis Imperfecta, (AI) |
| Other | Congenital Keratoconjunctivitis Sicca and Ichthyosiform Dermatosis, (CKCSID) |
| Other | Dental Hypomineralisation; mutation originally found in Border Collie |
| Other | Narcolepsy; mutation originally found in Dachshund |
| Other | Narcolepsy; mutation originally found in Doberman Pinscher |
| Other | Narcolepsy; mutation originally found in Labrador Retriever |
| Other | Persistent Müllerian Duct Syndrome, (PMDS); mutation originally found in Miniature Schnauzer |
| Other | Primary Ciliary Dyskinesia, (PCD) |
| Renal | Cystinuria Type I-A; mutation originally found in Newfoundland Dog |
| Renal | Cystinuria Type II-A; mutation originally found in Australian Cattle Dog |
| Renal | Cystinuria, Type II-B; mutation originally found in Miniature Pinscher |
| Renal | Fanconi Syndrome |
| Renal | Hyperuricosuria, (HUU) |
| Renal | Polycystic Kidney Disease in Bull Terriers, (BTPKD) |
| Renal | Primary Hyperoxaluria, (PH); mutation originally found in Coton de Tulear |
| Renal | Protein Losing Nephropathy, (PLN); NPHS1 gene variant |
| Renal | Renal Cystadenocarcinoma and Nodular Dermatofibrosis, (RCND) |
| Renal | X-Linked Hereditary Nephropathy, (XLHN) |
| Renal | X-Linked Hereditary Nephropathy, (XLHN); mutation originally found in Navasota Dog |
| Renal | Xanthinuria, Type 1a; mutation originally found in mixed-breed dogs |
| Renal | Xanthinuria, Type 2a; mutation originally found in Toy Manchester Terrier |
| Renal | Xanthinuria, Type 2b; mutation originally found in Cavalier King Charles Spaniel and English Cocker Spaniel |
| Skeletal | Chondrodysplasia; mutation originally found in Norwegian Elkhound and Karelian Bear Dog |
| Skeletal | Cleft Palate; Cleft Lip and Palate with Syndactyly; ADAMTS20 gene mutation originally found in Nova Scotia Duck Tolling Retriever |
| Skeletal | Cleft Palate; DLX6 gene mutation originally found in Nova Scotia Duck Tolling Retriever |
| Skeletal | Craniomandibular Osteopathy, (CMO); mutation associated with terrier breeds |
| Skeletal | Hereditary Vitamin D-Resistant Rickets, (HVDRR) |
| Skeletal | Oculoskeletal Dysplasia 2 or Dwarfism-Retinal Dysplasia 2, (OSD2) |
| Skeletal | Osteochondrodysplasia; mutation originally found in Miniature Poodle |
| Skeletal | Osteogenesis Imperfecta, (OI); mutation originally found in Beagle |
| Skeletal | Osteogenesis Imperfecta, (OI); mutation originally found in Dachshund |
| Skeletal | Skeletal Dysplasia 2, (SD2) |
| Skeletal | Spondylocostal Dysostosis |
| Skeletal | Van den Ende-Gupta Syndrome, (VDEGS) |
